# Comparative genomics to investigate the emergence of community-associated methicillin-resistant Staphylococcus aureus (CA-MRSA) USA300 clone in Geneva, Switzerland

**DOI:** 10.1186/2047-2994-4-S1-P197

**Published:** 2015-06-16

**Authors:** SM Diene, E Von Dach, C Fankhauser, E-J Bonetti, J Schrenzel, S Harbarth, P François

**Affiliations:** 1 Clinical Microbiology and Genomic Research Laboratories, Geneva, Switzerland; 2 Infection Control Program, Univ. Hospitals, Geneva, Switzerland

## Introduction

Molecular epidemiological surveys of CA-MRSA revealed a wide diversity of genetic backgrounds with only sporadic identification of USA300 isolates during the period 1994-2012.

## Objectives

We conducted a comparative genomics approach to trace origin, spreading and diversity of CA-MRSA USA300 clones accounting for 50% of CA-MRSA isolates identified in 2013.

## Methods

Solexa-Illumina was used for whole genome sequencing (WGS) of all USA300 isolated in 2013. Comparative genomics identified genomic alterations in this “clonal” population. All features including single-nucleotide polymorphisms (SNPs), ACME gene cluster, SCC *mec* structure, and mobile elements were documented and enriched with patient information. Published genomes of USA300 were used for comparison purposes and for investigating the relationship between isolates.

## Results

From 1994 to 2005, only 4 USA300 strains were identified in our institution. In 2013, among the 46 cases of CA-MRSA, USA300 were found in 22 patients (12 clinical infections, 10 cases of asymptomatic carriage). WGS allowed identifying two groups: (i) ACME positive (n=12) and (ii) ACME negative (n=10). In contrast to ACME-neg, the ACME-pos strains were resistant to ciprofloxacin and erythromycin. Comparison with a reference genome revealed that the ACME-pos group was more homogeneous that ACME-neg showing reduced genome plasticity. Two clusters of 2 strains were identified describing familial transmission events. The vast majority of ACME neg strains were isolated from patients traveling to South America in the 12 last months. SNP position allowed tracing the geographical origin of strains and to observe that ACME-neg group is composed by strains harboring a SCC *mec* IVc element.

## Conclusion

In 2013, we observe a sudden and worrisome increase in CA-MRSA USA300 isolates in Geneva. WGS showed that acquisition of mobile elements and smaller genomic alterations are signatures of strain origin, probably related to antibiotic utilization. Our epidemiology is rapidly changing. Considering that most of USA300 result from importation events, its emergence coincides probably with loss of fitness of ancient clones.

## Disclosure of interest

None declared.

